# Evaluation of Racial Disparities in Quality of Care for Patients With Gastrointestinal Tract Cancer Treated With Surgery

**DOI:** 10.1001/jamanetworkopen.2022.5664

**Published:** 2022-04-04

**Authors:** Baylee F. Bakkila, Daniel Kerekes, Marcella Nunez-Smith, Kevin G. Billingsley, Nita Ahuja, Karen Wang, Carol Oladele, Caroline H. Johnson, Sajid A. Khan

**Affiliations:** 1 currently a medical student at Yale School of Medicine, New Haven, Connecticut; 2 Department of Surgery, Yale University School of Medicine, New Haven, Connecticut; 3 Equity Research and Innovation Center, Section of General Internal Medicine, Yale School of Medicine, New Haven, Connecticut; 4 Division of Surgical Oncology, Department of Surgery, Yale School of Medicine, New Haven, Connecticut; 5 Equity Research and Innovation Center, Yale School of Medicine, New Haven, Connecticut; 6 Department of Environmental Health Sciences, Yale School of Public Health, Yale University, New Haven, Connecticut

## Abstract

**Importance:**

Racial disparities have been demonstrated in many facets of health care, but a comprehensive understanding of who is most at risk for substandard surgical care of gastrointestinal tract cancers is lacking.

**Objective:**

To examine racial disparities in quality of care of patients with gastrointestinal tract cancers.

**Design, Setting, and Participants:**

This retrospective cohort study of patients with gastrointestinal tract cancer included the US population as captured in the National Cancer Database with a diagnosis from January 1, 2004, to December 31, 2017. Participants included 565 124 adults who underwent surgical resection of gastrointestinal tract cancers. Data were analyzed from June 21 to December 23, 2021.

**Exposures:**

Race and site of cancer.

**Main Outcomes and Measures:**

Oncologic standard of care, as defined by negative resection margin, adequate lymphadenectomy, and receipt of indicated adjuvant chemotherapy and/or radiotherapy.

**Results:**

Among 565 124 adult patients who underwent surgical resection of a gastrointestinal tract cancer, 10.9% were Black patients, 83.5% were White patients, 54.7% were men, and 50.7% had Medicare coverage. The most common age range at diagnosis was 60 to 69 years (28.5%). Longer median survival was associated with negative resection margins (87.3 [IQR, 28.5-161.9] months vs 22.9 [IQR, 8.8-69.2] months; *P* < .001) and adequate lymphadenectomies (80.7 [IQR, 25.6 to not reached] months vs 57.6 [IQR, 17.7-153.8] months; *P* < .001). After adjustment for covariates, Black patients were less likely than White patients to have negative surgical margins overall (odds ratio [OR], 0.96 [95% CI, 0.93-0.98]) and after esophagectomy (OR, 0.71 [95% CI, 0.58-0.87]), proctectomy (OR, 0.71 [95% CI, 0.66-0.76]), and biliary resection (OR, 0.75 [95% CI, 0.61-0.91]). Black patients were also less likely to have adequate lymphadenectomy overall (OR, 0.89 [95% CI, 0.87-0.91]) and after colectomy (OR, 0.89 [95% CI, 0.87-0.92]), esophagectomy (OR, 0.72 [95% CI, 0.63-0.83]), pancreatectomy (OR, 0.90 [95% CI, 0.85-0.96]), proctectomy (OR, 0.93 [95% CI, 0.88-0.98]), proctocolectomy (OR, 0.90 [95% CI, 0.81-1.00]), and enterectomy (OR, 0.71 [95% CI, 0.65-0.79]). Black patients were more likely than White patients not to be recommended for chemotherapy (OR, 1.15 [95% CI, 1.10-1.21]) and radiotherapy (OR, 1.49 [95% CI, 1.35-1.64]) because of comorbidities and more likely not to receive recommended chemotherapy (OR, 1.68 [95% CI, 1.55-1.82]) and radiotherapy (OR, 2.18 [95% CI, 1.97-2.41]) for unknown reasons.

**Conclusions and Relevance:**

These findings suggest that there are significant racial disparities in surgical care of gastrointestinal tract cancers. Black patients are less likely than White patients to receive standard of care with respect to negative surgical margins, adequate lymphadenectomies, and use of adjuvant therapies. Both system- and physician-level reforms are needed to eradicate these disparities in health care.

## Key Points

**Question:**

Are there racial disparities in the quality of surgical care among patients with gastrointestinal tract cancers?

**Findings:**

In this cohort study of 565 124 patients with gastrointestinal tract cancer who underwent surgical resection, Black patients had lower rates of negative margins, adequate lymphadenectomy, and receipt of adjuvant therapy compared with White patients. Receiving care incongruent with current standards was associated with decreased survival, and compromised survival was greatest for Black patients.

**Meaning:**

These findings suggest that racial disparities in surgical care of gastrointestinal tract cancers exist, and that system- and physician-level changes are needed to address and eradicate the root causes of these disparities.

## Teaser

This cohort study examines whether racial disparities exist in treatment received and in survival among patients undergoing surgical intervention with curative intent for gastrointestinal tract cancer.

## Introduction

Racial and ethnic disparities have long been reported in the health care system, and the COVID-19 pandemic has further highlighted these inequities in medicine with disproportionately higher rates of both infection and death in Black and Hispanic patients.^1,2,3^ Cancer treatment has not been immune to these incongruities, with racial and ethnic disparities consistently observed in survival outcomes.^4,5^ Although national treatment guidelines exist, differences in care of patients based on sociodemographic features still persist and may contribute to these observed survival inequities.^6,7^

Gastrointestinal tract cancer comprises a large proportion of cancer burden. It accounts for 26% of cancers globally and 35% of all cancer-related mortality, with increasing age-standardized incidence of colon, rectal, liver, intrahepatic bile duct, stomach, and pancreatic cancers projected in the US during the coming decades.^8,9^ Black patients have a disproportionate gastrointestinal tract cancer burden compared with White patients, with a 19% excess risk of cancer death for men and 13% excess risk for women.^10,11^ Although innovative treatments exist, evidence suggests that inequities in access to quality care may explain part of the disproportionate incidence of and mortality due to cancer in Black patients.^10^

Evidence shows that Black patients are less likely than White patients to receive any surgical intervention for their gastrointestinal tract cancer, including esophageal and colorectal cancers.^12,13,14^ This observed difference in access to effective treatment has been associated with lower survival rates among Black patients compared with White patients.^13,15^ In 2003, the Institute of Medicine published a comprehensive review of treatment disparities and potential causes, concluding that patients in racial minority groups were receiving lower quality of health care.^16^ They called on clinicians, patients, and society at large to address these inequities. Eighteen years later, the goal of this study is to provide an update to these findings as they relate to surgical care of gastrointestinal tract cancer in a cohort of patients diagnosed and treated after the Institute of Medicine’s report and call to action.

Although some investigators have quantified existing disparities in access to surgery, few have examined disparities in quality of care among those who underwent surgical resection and why these disparities may exist. In this study, we aim to examine whether there are racial disparities in cancer care for patients undergoing surgical intervention with curative intent. Accordingly, we examined the likelihood of negative resection margin, site-specific adequate lymphadenectomy, and receipt of adjuvant therapy by race in 12 gastrointestinal tract cancers as proxies for quality of surgical and perioperative cancer care received by a patient. We also looked at racial differences in survival and in reasons for omission of adjuvant therapy. Ultimately, we aimed to better understand the mechanism behind worse survival outcomes among Black patients with gastrointestinal tract cancer and the possible contribution of variations in quality of surgical and perioperative care.

## Methods

### Data Source and Study Sample

We used data from the National Cancer Database (NCDB) 2017 Participant Use File for this cohort study. The NCDB is a joint project between the American College of Surgeons and the American Cancer Society sourced from 1500 Commission on Cancer–accredited facilities, representing more than 70% of newly diagnosed cancer cases in the US. Adult participants (aged ≥18 years) who were diagnosed with a malignant neoplasm of the gastrointestinal tract from January 1, 2004, to December 31, 2017, and underwent surgical resection of their primary cancer site were included in our analysis. Exclusion criteria included missing demographic and clinical data as well as unknown surgery type or local tumor destruction, rather than surgical resection. Missing variables were assumed to be missing completely at random because all data were extracted from the medical record by trained clinical reviewers at participating institutions. Operations described as local tumor excisions were included in resection margin analysis but omitted from lymphadenectomy analysis. This study followed the Strengthening the Reporting of Observational Studies in Epidemiology (STROBE) reporting guideline. Because the NCDB is a deidentified database, this research study did not qualify as human subjects research and was therefore not reviewed in accordance with Yale University Institutional Review Board guidelines.

### Outcome Measures

#### Resection Margins

Resection margins were noted to be negative if all surgical resection margins were grossly and microscopically negative. Patients who had margins with residual tumor that was macroscopic, microscopic, or not otherwise specified were classified as having positive margins.

#### Adequate Lymphadenectomy

Adequate lymphadenectomy was defined according to standards set by the National Comprehensive Cancer Network, American Joint Committee on Cancer staging manuals, or expert consensus based on quality metric studies spanning more than 2 decades. Number of lymph nodes examined, as captured by the NCDB, was recoded into a binary adequate or inadequate variable based on the standard for each respective cancer site. Having 12 or more lymph nodes examined was considered adequate for cancers of the colon, rectosigmoid, rectum, and anus.^17,18^ For the esophagus, pancreas, and stomach, a cutoff of 15 or more lymph nodes was used; for the small intestine, 8 or more lymph nodes; and for the gallbladder, 6 or more lymph nodes.^19,20,21,22,23,24^ Liver, other biliary, and peritoneal primary site cancers were not included in the lymphadenectomy analysis.

#### Adjuvant Therapy

We used a variable within the NCDB that captures whether a patient was recommended to receive adjuvant therapy (chemotherapy or radiotherapy), if they received it, and if not, why it was omitted from the treatment regimen. Reasons included refused, not administered due to comorbidities, or unknown.

#### Survival Outcomes

Individual time from diagnosis to death or last contact is a reported variable in the NCDB. Association of survival outcomes with resection margins and adequate lymphadenectomy were calculated.

### Demographic, Socioeconomic, and Clinical Covariates

From the NCDB Participant User File, we extracted demographic and treatment site information, including age at diagnosis, sex, race, ethnicity, primary payer at the time of diagnosis, and facility type. In the NCDB, race and ethnicity are collected from medical records, electronic medical record billing records, or self-reported by the patient, using standardized options for both race and ethnicity, including the category “other.” The American Indian category included Aleutian and Inuit patients, and the Asian and Pacific Islander groups were collapsed from appropriate NCDB categories. Age at diagnosis was extracted as a continuous variable and grouped by decade.

To control for cancer characteristics, we also extracted primary tumor and patient information, including organ of primary site (grouped by *International Statistical Classification of Diseases and Related Health Problems, Tenth Revision* codes), grade or differentiation, American Joint Committee on Cancer clinical stage group, and Charlson Comorbidity Index scores. Charlson Comorbidity Index scores capture comorbidity based on 15 weighted categories of patient characteristics and diagnoses, with higher scores indicating greater comorbidity and risk of mortality.^25^

### Statistical Analysis

Data were analyzed from June 21 to December 23, 2021. Analyses focused on comparisons between Black and White patients because Black patients have a disproportionate burden and lower survival rates of gastrointestinal tract cancers compared with White patients. Descriptive statistics were run to characterize the patient population in aggregate and stratified by race. We fit multivariable logistic regressions to examine how race was associated with odds of negative resection margin and adequate lymphadenectomy during resection. Four multivariable regressions were used to evaluate the robustness of the association between surgical outcome metric and race to better understand the role of covariate adjustment. Model 1 was unadjusted; model 2, adjusted for sex, age group, race, and organ site; model 3, additionally adjusted for primary payer, facility type, and Charlson Comorbidity Index score; and model 4, adjusted for model 2 and 3 covariates and clinical TNM stage group and grade. In organ-specific analyses and Cox proportional hazards regressions, model 2 was used to control for the most important covariates while avoiding inclusion of variables that may be in the causal pathway for racially disparate treatment outcomes.

Regression models were run in aggregate and individually by cancer site. We set α = .05, and a 2-sided *P* < .05 indicated statistical significance. We performed χ^2^ tests of independence and odds ratio (OR) calculations to examine the association between race and use, omission, and reason for omission of recommended adjuvant therapy. Hypothesis tests were 2-sided. Cox proportional hazards regressions and hazard ratios (HRs) were calculated to examine the associations between surgical outcomes and race for all cancer types and by organ site. All analyses were conducted in SPSS Statistics, version 28.0.0.0 (IBM Corporation).

## Results

**Figure 1.**
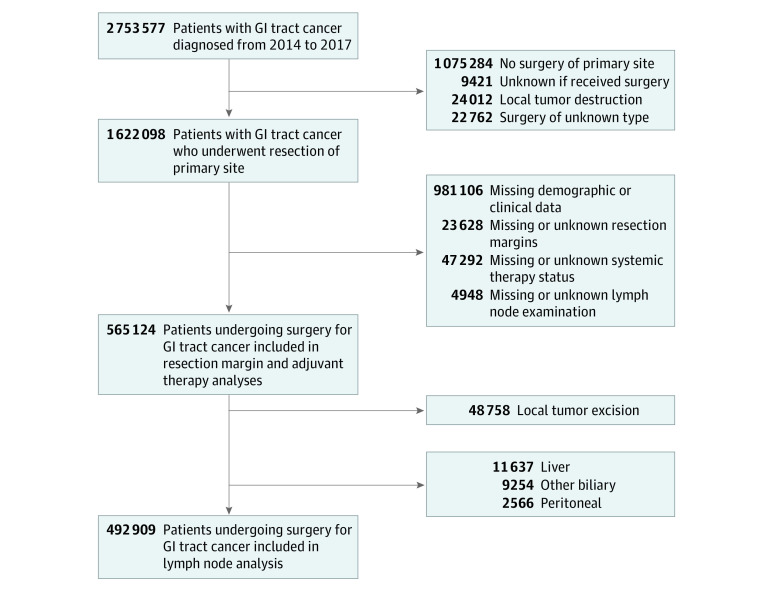
Inclusion Criteria for Analysis GI indicates gastrointestinal.

A total of 565 124 patients with gastrointestinal tract cancer met our inclusion criteria. The entire study population was used for resection margin analysis, whereas subsets were used for lymphadenectomy and adjuvant therapy analyses (Figure 1). There was a male predominance in the study population, with 309 027 men (54.7%) and 256 097 women (45.3%) (eTable 1 in the Supplement). The majority were White (472 133 [83.5%]), non-Hispanic (510 580 [90.3%]) patients; 1909 (0.3%) were American Indian, 19 431 (3.4%) were Asian, 61 750 (10.9%) were Black, 921 (0.2%) were Pacific Islander, 4544 (0.8%) were other race or ethnicity, and 4436 (0.8%) were of unknown race or ethnicity. The most common primary site was the colon (44.9%), and Medicare was the most common primary payer (50.7%) (eTable 1 in the Supplement). The most common age range at diagnosis was 60 to 69 years (28.5%), and the most common facility type was a comprehensive community cancer program (39.8%). Negative surgical resection margins were observed in 88.5% of patients and adequate lymphadenectomy was observed in 71.2% of patients.

### Resection Margins

**Figure 2.**
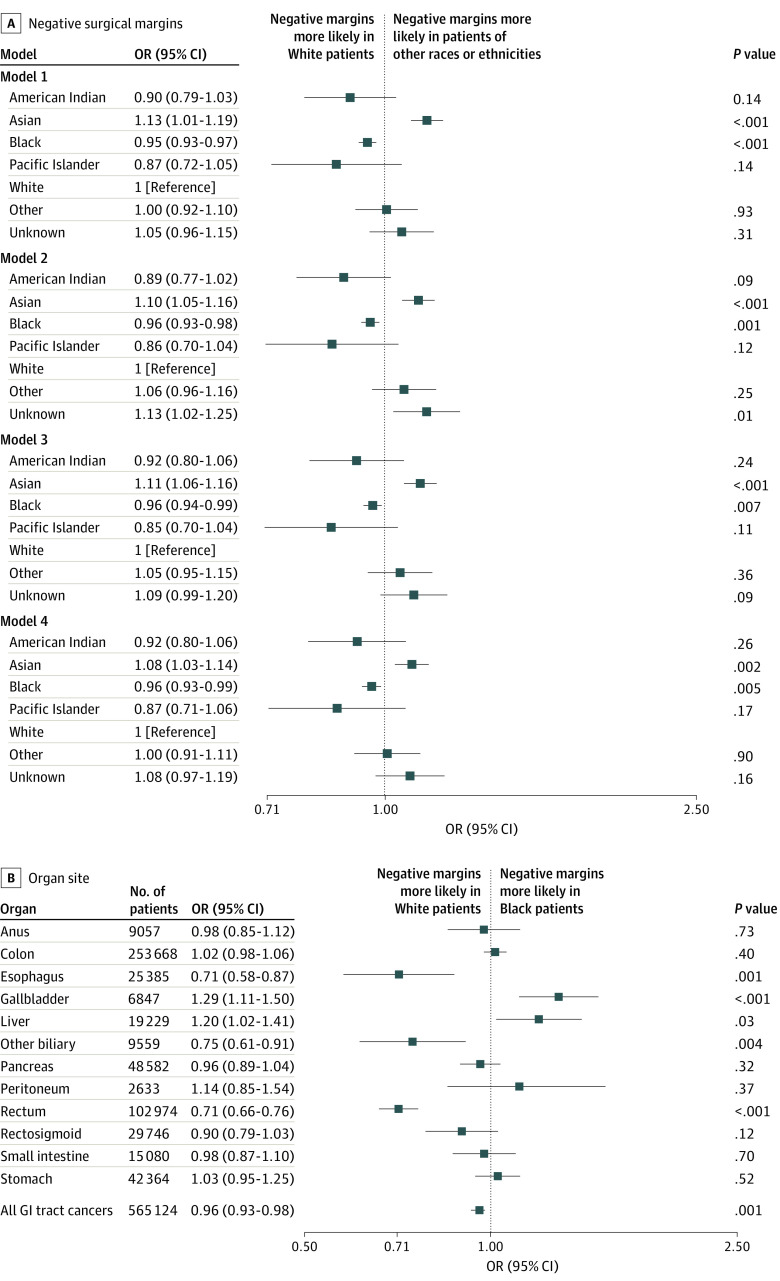
Odds Ratios (ORs) of Negative Surgical Margins by Race A, Odds ratios of negative surgical margins by race in 4 models. Model 1 was unadjusted; model 2, adjusted for sex, age group, race, and organ site; model 3, adjusted for covariates in model 2 and primary payer, facility type, and Charlson Cormorbidity Index; and model 4, adjusted for covariates in models 2 and 3 and clinical TNM stage group and cancer grade. B, Odds ratios of Black patients having negative resection margins compared with White patients were separated by organ site and controlled for covariates in model 2. GI indicates gastrointestinal.

In model 1 unadjusted analysis, we found that Black patients were less likely to have negative resection margins compared with White patients (OR, 0.95 [95% CI, 0.93-0.97]) (Figure 2A). Asian patients were more likely to have negative margins (OR, 1.13 [95% CI, 1.01-1.19]). In each subsequent logistic regression (models 2-4), we included numerous covariates in a stepwise fashion. The 4% disparity in negative resection margin for Black patients was observed in all models (OR for model 2, 0.96 [95% CI, 0.93-0.98]; OR for model 3, 0.96 [95% CI, 0.94-0.99]; and OR for model 4, 0.96 [95% CI, 0.93-0.99]).

When applying model 2 to individual cancers, differences in likelihood of negative resection margin varied according to race and ethnicity and site. Black patients were 29% more likely than White patients to have negative resection margins for gallbladder cancer (OR, 1.29 [95% CI, 1.11-1.50]) and 29% less likely after esophagectomy (OR, 0.71 [95% CI, 0.58-0.87]) (Figure 2B). Compared with White patients, Black patients were 25% less likely to have negative resection margins in other biliary site resections (OR, 0.75 [95% CI, 0.61-0.91]) and 29% less likely in proctectomies (OR, 0.71 [95% CI, 0.66-0.76]).

### Adequate Lymphadenectomy

**Figure 3.**
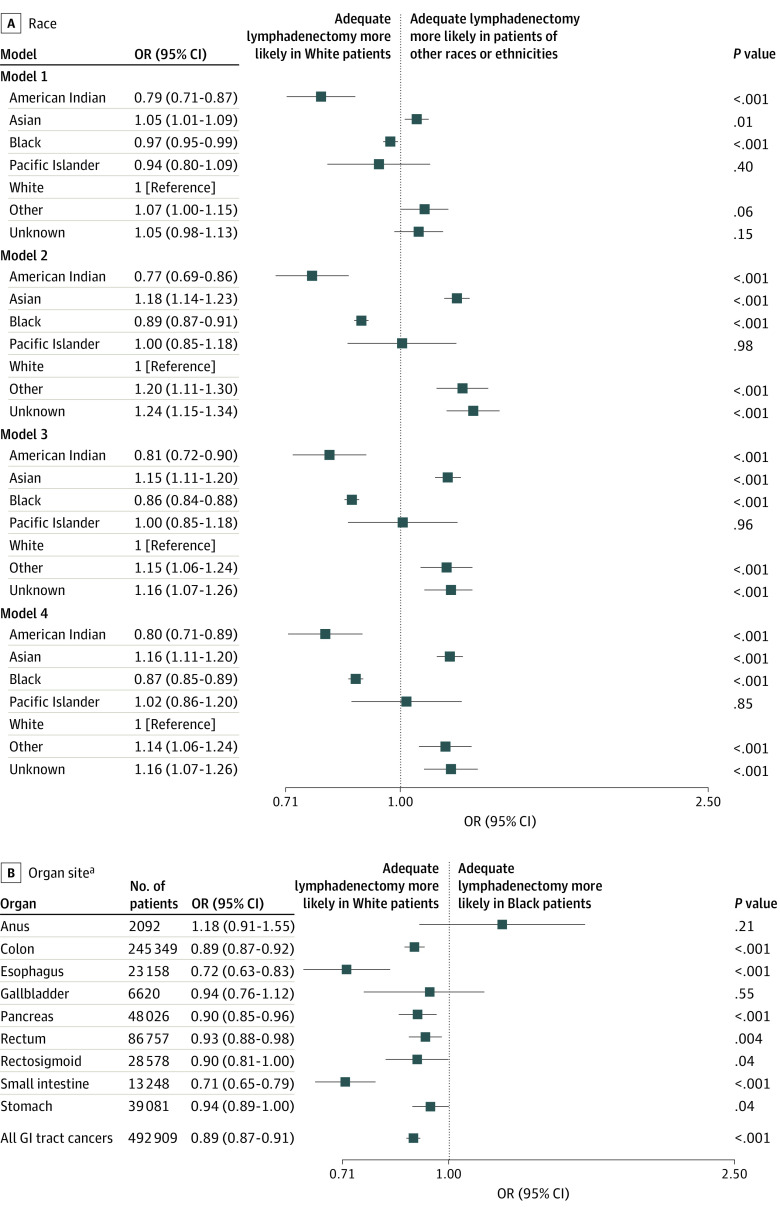
Odds Ratios (ORs) of Adequate Lymphadenectomy by Race A, Odds ratios of adequate lymph node removal by race in 4 models. Model 1 was unadjusted; model 2, adjusted for sex, age group, race, and organ site; model 3, adjusted for covariates in model 2 and primary payer, facility type, and Charlson Cormorbidity Index; and model 4, for covariates in models 2 and 3 and clinical TNM stage group and cancer grade. B, Odds ratios of Black patients receiving adequate lymph node removal compared with White patients were separated by organ site and controlled for covariates in model 2. ^a^Model 2.

Black patients were 3% less likely to have adequate lymphadenectomy compared with White patients in an unadjusted model (model 1 OR, 0.97 [95% CI, 0.95-0.99]) (Figure 3A). American Indian patients were also less likely to have adequate node examination when compared with White patients (OR, 0.79 [95% CI, 0.71-0.87]), whereas Asian patients were 5% more likely (OR, 1.05 [95% CI, 1.01-1.09]). With the inclusion of covariates in additional models, the magnitude of disparity between Black and White patients was increased from 11% to 14% (OR for model 2, 0.89 [95% CI, 0.87-0.91]; OR for model 3, 0.86 [95% CI, 0.84-0.88]; and OR for model 4, 0.87 [95% CI, 0.85-0.89]).

With respect to individual cancer types, differences in likelihood of adequate lymphadenectomy ranged from 6% less likely for Black patients than White patients undergoing gastrectomy (OR, 0.94 [95% CI, 0.89-1.00]) to 29% less likely after enterectomy (OR, 0.71 [95% CI, 0.65-0.79]) (Figure 3B). Compared with White patients, Black patients were less likely to have adequate lymphadenectomy after esophagectomy (OR, 0.72 [95% CI, 0.63-0.83]), colectomy (OR, 0.89 [95% CI, 0.87-0.92]), proctocolectomy (OR, 0.90 [95% CI, 0.81-1.00]), proctectomy (OR, 0.93 [95% CI, 0.88-0.98]), and pancreatectomy (OR, 0.90 [95% CI, 0.85-0.96]).

### Adjuvant Therapy

In unadjusted analysis and when compared with White patients, Black patients were 8% less likely to receive chemotherapy (OR, 0.92 [95% CI, 0.90-0.93]) and 35% less likely to receive radiotherapy (OR, 0.65 [95% CI, 0.63-0.66]) (Table). Omission of recommended adjuvant treatment was also more common in Black patients for both chemotherapy (OR, 1.15 [95% CI, 1.12-1.19]) and radiotherapy (OR, 1.48 [95% CI, 1.40-1.58]). The proportion of patients who refused recommended adjuvant therapy did not differ significantly by race (OR for chemotherapy, 1.01 [95% CI, 0.97-1.06]; OR for radiotherapy, 1.01 [95% CI, 0.91-1.12]) (Table). However, Black patients were 15% more likely than White patients not to be administered chemotherapy (OR, 1.15 [95% CI, 1.10-1.21]) and 49% more likely not to be administered radiotherapy (OR, 1.49 [95% CI, 1.35-1.64]) owing to comorbidities. Black patients were also 68% more likely not to receive recommended chemotherapy (OR, 1.68 [95% CI, 1.55-1.82]) and 118% more likely not to receive recommended radiotherapy (OR, 2.18 [95% CI, 1.97-2.41]) for unknown reasons.

**Table.** Use or Omission of Chemotherapy and Radiotherapy Among Black and White Patients

Adjuvant treatment	Black patients	White patients	OR (95% CI) for Black compared with White patients	*P* value
**Overall adjuvant treatment**				
Chemotherapy				
No. of patients included in analysis	59 741	459 814	NA	NA
No. (%) receiving	28 839 (48.3)	231 702 (50.4)	0.92 (0.90-0.93)	<.001
Radiotherapy				
No. of patients included in analysis	60 742	465 262	NA	NA
No. (%) receiving	10 780 (17.7)	116 202 (25.0)	0.65 (0.63-0.66)	<.001
**Omission of recommended adjuvant treatment**				
Chemotherapy				
No. of patients included in the analysis	34 165	269 321	NA	NA
All reasons, No. (%)	5326 (15.6)	37 619 (14.0)	1.15 (1.12-1.19)	<.001
Refused, No. (%)	2332 (6.8)	17 991 (6.7)	1.01 (0.97-1.06)	.64
Comorbidity contraindication, No. (%)	1883 (5.5)	13 414 (5.0)	1.15 (1.10-1.21)	<.001
Unknown, No. (%)	622 (1.8)	2943 (1.1)	1.68 (1.55-1.82)	<.001
Radiotherapy				
No. of patients included in the analysis	12 121	125 962	NA	NA
All reasons, No. (%)	1341 (11.1)	9750 (7.7)	1.48 (1.40-1.58)	<.001
Refused, No. (%)	395 (3.3)	4079 (3.2)	1.01 (0.91-1.12)	.90
Comorbidity contraindication, No. (%)	484 (4.0)	3420 (2.7)	1.49 (1.35-1.64)	<.001
Unknown, No. (%)	462 (3.8)	2251 (1.8)	2.18 (1.97-2.41)	<.001

Abbreviations: NA, not applicable; OR, odds ratio.

### Survival Outcomes

**Figure 4.**
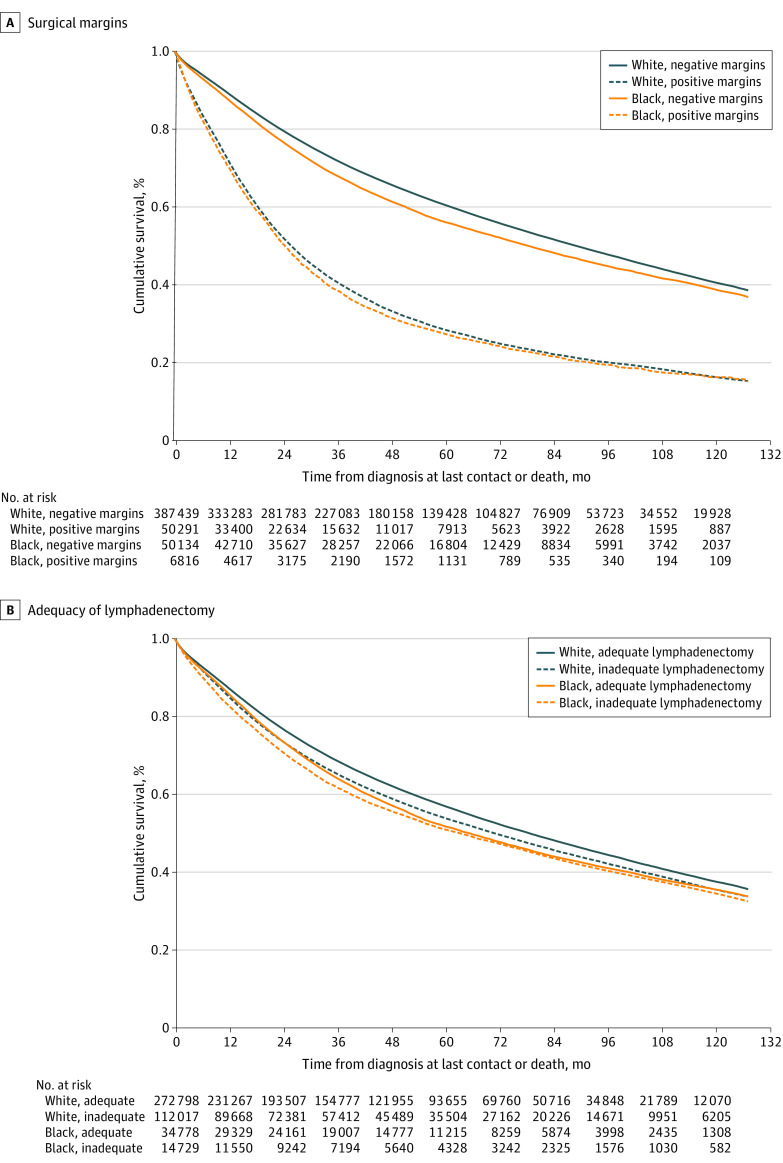
Survival Functions by Outcome Metric of Interest and Race Cox proportional hazards regression curves (adjusted for age group, sex, race, and organ site of cancer [model 2]) and hazard ratios (HRs) stratified by White or Black race. For surgical resection margins (A), HR for White race and positive margins was 2.54 (95% CI, 2.51-2.57); for Black race and negative margins, 1.12 (95% CI, 1.10-1.14); and for Black race and positive margins, 2.65 (95% CI, 2.57-2.71), with White race and negative margins as the reference (*P* < .001). For lymph node removal (B), HR for White race and inadequate lymphadenectomy was 1.10 (95% CI, 1.09-1.11); for Black race and adequate lymphadenectomy, 1.13 (95% CI, 1.11-1.15); and for Black race and inadequate lymphadenectomy, 1.20 (95% CI, 1.17-1.23), with White race and adequate lymphadenectomy as the reference category (*P* < .001).

Median follow-up for the entire cohort was 41.0 (IQR, 19.4-72.0) months. Negative resection margins were associated with a longer median survival than positive margins (87.3 [IQR, 28.5-161.9] months vs 22.9 [IQR, 8.8-69.2] months; *P* < .001). Compared with White patients with negative resection margins, Black patients with negative resection margins had increased risk of death (HR, 1.12 [95% CI, 1.10-1.14]) (Figure 4A). White patients with positive resection margins had increased risk of death (HR, 2.54 [95% CI, 2.51-2.57]), as did Black patients with positive resection margins (HR, 2.65 [95% CI, 2.57-2.71]). Black patients with negative resection margins after proctocolectomies had a 35% higher risk of death than white patients with negative resection margins (HR, 1.35 [95% CI, 1.26-1.45]), a 27% higher risk after proctectomies with negative resection margins (HR, 1.27 [95% CI, 1.08-1.50]), and a 24% higher risk after enterectomies with negative resection margins (HR, 1.24 [95% CI, 1.13-1.36]) (eTable 2 in the Supplement).

Adequate lymphadenectomy was associated with a longer median survival than inadequate lymphadenectomy (80.7 [IQR, 25.6 to not reached] months vs 57.6 [IQR, 17.7-153.8] months; *P* < .001). Overall median follow-up was 40.7 (IQR, 19.0-72.0) months. Compared with White patients with adequate lymphadenectomy, Black patients with adequate lymphadenectomy had an increased risk of death (HR, 1.13 [95% CI, 1.11-1.15]), as did White patients with inadequate lymphadenectomy (HR, 1.10 [95% CI, 1.09-1.11]) and Black patients with inadequate lymphadenectomy (HR, 1.20 [95% CI, 1.17-1.23]) (Figure 4B).

## Discussion

In this cohort study of more than half a million patients with gastrointestinal tract cancer undergoing surgery with curative intent, Black patients were more likely than White patients to receive substandard cancer care. Black patients had a 4% lower odds of negative resection margin and 11% lower odds of adequate lymphadenectomy (model 2). In addition, Black patients were 15% and 49% more likely than White patients to not receive indicated adjuvant chemotherapy and radiotherapy, respectively, owing to comorbidities, and 68% and 118% more likely, respectively, not to receive these therapies for unknown reasons. These observed differences in care are concerning in their own right, but they may also contribute to the worse survival outcomes that have been reported among Black patients with gastrointestinal tract cancer.^4,5^ Accordingly, disparate oncologic treatment for Black patients, likely working in conjunction with other disparities in lived experience, were shown to exacerbate survival differences associated with surgical outcomes.

Prior research^12,13,14,15^ has demonstrated racial disparities in both receipt of surgery and overall survival for Black patients with cancer but understates the problematic disparities in care between Black and White patients by limiting analysis to small clinical stage subgroups. In addition, they fail to look at treatment metrics beyond receipt of surgery. Our study fills these gaps by providing a comprehensive analysis of these disparities with 3 critical treatment metrics among 12 cancer types. Among patients who underwent surgical intervention, Black patients with gastrointestinal tract cancer appear to receive worse care than their White counterparts before, during, and after oncologic surgical resection. In addition, our organ-specific analyses permit comparison across primary sites and indicate those with the most severe disparities. For gallbladder cancer, resection margin outcomes were better for Black patients than White patients, although this outlier was observed in a small sample size.

Outcomes regarding both resection margins and lymphadenectomy are determined by a myriad of factors, including patient anatomy, hospital and pathology department capacitance, and a surgeon’s technical abilities and decision-making. In fact, the magnitude of the disparities for age and facility type were larger than that for race and ethnicity in our adjusted models (eFigures 1 and 2 in the Supplement). Although factors such as type of treatment center and hospital volume have been reported previously to correlate with greater lymph node removal and likelihood of negative resection margins,^26,27^ we observed racial disparities in our study when controlling for these covariates. Of note, a sensitivity analysis that additionally adjusted for facility volume and racial composition of patient population showed that inclusion of these variables did not change the significance of our findings. Systemic barriers beyond facility type may be incompletely controlled for by our analyses and may lead to systematic disadvantages for Black patients, such as higher disease burden at diagnosis, thereby contributing to our observed disparities.^28,29^

The presence of disparities at this stage of care, and after controlling for clinical factors, indicates the need to better understand lapses in the system, such as when and why surgical margins are not checked intraoperatively and when and why not all collected lymph nodes are properly dissected and examined. Implicit bias of clinicians, for example, has been correlated with racial differences in management of metastatic cancer–related pain and shorter, less comprehensible treatment discussions with patients with cancer.^30,31^ Identification of possible biases, such as time spent on lymph node dissections and unrecognized cultural biases by clinicians and health care systems, is needed to better understand racial disparities in care. Subsequently, appropriate interventions should be made, such as additional quality improvement measures or further bias training to ensure standard of care is given to every patient with cancer, regardless of race or ethnicity.

The decision to use adjuvant therapy is multifocal, with patients, families, and clinicians contributing. Although Black patients were less likely to be treated with adjuvant therapy overall and less likely to receive it when recommended, they were just as likely to refuse recommended therapy as White patients, indicating that patient decision-making largely does not differ by race in this instance. Further research probing potential biases in perception of comorbidities by clinicians as well as system factors, such as access to transportation to adjuvant therapy, is needed to understand and address this treatment disparity. In addition, exploration into the currently unknown reasons for omission of recommended therapy will be essential to addressing disparities in cancer outcomes.

### Limitations

Our study has some limitations. First, the NCDB lacks individual-level factors, such as body mass index, that may contribute to disparities in surgical care. However, we were able to use the Charlson Comorbidity Index in our model to control for comorbid conditions. Second, our survival analyses look at time from diagnosis, as opposed to time from surgery. However, we believe that using survival starting from diagnosis allows us to capture more elements of potential racial bias in the treatment pathway. For example, later diagnosis or treatment initiation due to race or ethnicity may lead to poorer surgical outcomes and survival and are therefore important to capture in our disparity analysis. Third, we restricted analysis to include only patients undergoing surgery, but racial and ethnic disparities in access to surgery itself have been previously reported,^12,13,14^ indicating our study likely underestimates the differences in care between Black and White patients with gastrointestinal tract cancer. Finally, our data do not capture cancer treatment and outcomes during the COVID-19 pandemic, which may lead to underestimation of disparities given the added burden of the pandemic and interruptions to care, especially for patients in racial and ethnic minority groups.^1,2,3^

## Conclusions

The findings of this cohort study suggest that Black patients with gastrointestinal tract cancer are more likely than their White counterparts to receive surgical and adjuvant treatment that deviates from the standard of care. These findings suggest that potential lapses in the health care system by clinicians and hospital systems contribute to racial and ethnic differences in survival by way of treatment disparities. Our study also found treatment disparities for American Indian patients, warranting additional inquiry. Further research should aim to probe both system- and physician-level drivers of disparities within cancer care and address places of bias that allow for this inequitable treatment of both Black and American Indian patients.

## Notes

Supplement. **eTable 1.** Demographic and Clinical Characteristics of Patients **eTable 2.** Survival Analysis by Outcome Metric, Race, and Organ Site **eFigure 1.** Odds Ratios of Negative Surgical Margins With Covariates (Model 2) **eFigure 2.** Odds Ratios of Adequate Lymphadenectomy With Covariates (Model 2)
