# Supplementary material for: Evaluation of Racial Disparities in Quality of Care for Patients With Gastrointestinal Tract Cancer Treated With Surgery
Source: JAMA Netw Open. 2022 Apr 4;5(4):e225664. doi: 10.1001/jamanetworkopen.2022.5664 (PMC8980937; doi:10.1001/jamanetworkopen.2022.5664)
Supplement: Supplement. — eTable 1. Demographic and Clinical Characteristics of Patients eTable 2. Survival Analysis by Outcome Metric, Race, and Organ Site eFigure 1. Odds Ratios of Negative Surgical Margins With Covariates (Model 2) eFigure 2. Odds Ratios of Adequate Lymphadenectomy With Covariates (Model 2) [file jamanetwopen-e225664-s001.pdf]

Supplementary Online Content

Bakkila BF, Kerekes D, Nunez-Smith M, et al. Evaluation of racial disparities in quality of care for patients with gastrointestinal tract cancer treated with surgery. *JAMA Netw Open*. 2022;5(4):e225664. doi:10.1001/jamanetworkopen.2022.5664

eTable 1. Demographic and Clinical Characteristics of Patients

eTable 2. Survival Analysis by Outcome Metric, Race, and Organ Site

eFigure 1. Odds Ratios of Negative Surgical Margins With Covariates (Model 2)

eFigure 2. Odds Ratios of Adequate Lymphadenectomy With Covariates (Model 2)

This supplementary material has been provided by the authors to give readers additional information about their work.

© 2022 Bakkila BF et al. *JAMA Network Open*.

eTable 1: Demographic and Clinical Characteristics of Patients
Characteristics of gastrointestinal cancer patients in our study population, stratified by white or Black race. *P*-values of Chi-squared tests reported.

Characteristic	All Gastrointestinal Cancer Patients (%)	White Gastrointestinal Cancer Patients (%)	Black Gastrointestinal Cancer Patients (%)	*P*-value
**Demographic**	**N = 565,124**	**N = 472,133**	**N = 61,750**	
Sex				<0.001
Female	256,097 (45.3)	211,180 (44.7)	31,229 (50.6)	
Male	309,027 (54.7)	260,953 (55.3)	30,521 (49.4)	
Age at diagnosis				<0.001
40-49	51,303 (9.1)	40,170 (8.5)	7,262 (11.8)	
50-59	125,342 (22.2)	100,153 (21.2)	17,183 (27.8)	
60-69	161,269 (28.5)	133,593 (28.3)	18,584 (30.1)	
70-79	140,237 (24.8)	120,491 (25.5)	12,794 (20.7)	
80-89	76,951 (13.6)	68,593 (14.5)	5,360 (8.7)	
90+	10,022 (1.8)	9,133 (1.9)	567 (0.9)	
Race				<0.001
American Indian, Aleutian, or Eskimo	1,909 (0.3)	0 (0)	0 (0)	
Asian	19,431 (3.4)	0 (0)	0 (0)	
Black	61,750 (10.9)	0 (0)	61,750 (100.0)	
Pacific Islander	921 (0.2)	0 (0)	0 (0)	

© 2022 Bakkila BF et al. *JAMA Network Open*.

Characteristic	All Gastrointestinal Cancer Patients (%)	White Gastrointestinal Cancer Patients (%)	Black Gastrointestinal Cancer Patients (%)	*P*-value
White	472,133 (83.5)	472,133 (100.0)	0 (0)	
Other[1]	4,544 (0.8)	0 (0)	0 (0)	
Unknown	4,436 (0.8)	0 (0)	0 (0)	
Ethnicity				<0.001
Not Hispanic	510,580 (90.3)	425,932 (90.2)	59,041 (95.6)	
Hispanic	30,815 (5.4)	27,617 (5.8)	661 (1.1)	
Unknown	23,729 (4.2)	18,584 (3.9)	2,048 (3.3)	
Primary payor at diagnosis				<0.001
Not Insured	16,218 (2.9)	11,556 (2.4)	3,431 (5.6)	
Private Insurance	217,628 (38.5)	181,719 (38.5)	22,500 (36.4)	
Medicaid	31,494 (5.6)	20,988 (4.4)	6,873 (11.1)	
Medicare	286,468 (50.7)	247,446 (52.4)	27,088 (43.9)	
Other Government	6,213 (1.1)	4,834 (1.0)	834 (1.4)	
Unknown	7,103 (1.3)	5,590 (1.2)	1,024 (1.7)	
Facility Type				<0.001
Community Cancer Program	53,516 (9.5)	46,211 (9.8)	4,505 (7.3)	
Comprehensive Community Cancer Program	224,802 (39.8)	194,672 (41.2)	20,598 (33.4)	

© 2022 Bakkila BF et al. *JAMA Network Open*.

Characteristic	Gastrointestinal Cancer Patients (%)	White Gastrointestinal Cancer Patients (%)	Black Gastrointestinal Cancer Patients (%)	*P*-value
Academic/Research Program	212,303 (37.6)	168,785 (35.7)	28,071 (45.5)	
Integrated Network Cancer Program	74,503 (13.2)	62,465 (13.2)	8,576 (13.9)	
**Clinical**	**N = 565,124**			
Charlson-Deyo Score				<0.001
0	391,377 (69.3)	327,705 (69.4)	41,018 (66.4)	
1	121,882 (21.6)	101,193 (21.4)	14,320 (23.2)	
2	33,648 (6.0)	28,328 (6.0)	3,969 (6.4)	
>=3	18,217 (3.2)	14,907 (3.2)	2,443 (4.0)	
Primary Site				<0.001
Anus	9,057 (1.6)	7,824 (1.7)	1,001 (1.6)	
Colon	253,668 (44.9)	211,254 (44.7)	31,150 (50.4)	
Esophagus	25,385 (4.8)	23,660 (5.0)	962 (1.6)	
Gallbladder	6,847 (1.2)	5,355 (1.1)	999 (1.6)	
Liver	19,229 (3.4)	14,245 (3.0)	2,403 (3.9)	
Other Biliary	9,559 (1.7)	8,050 (1.7)	742 (1.2)	
Pancreas	48,582 (8.6)	41,497 (8.8)	4,796 (7.8)	

© 2022 Bakkila BF et al. *JAMA Network Open*.

Characteristic	All Gastrointestinal Cancer Patients (%)	White Gastrointestinal Cancer Patients (%)	Black Gastrointestinal Cancer Patients (%)	*P*-value
Peritoneum	2,633 (0.5)	2,303 (0.5)	196 (0.3)	
Rectum	102,974 (18.2)	88,340 (18.7)	8,712 (14.1)	
Rectosigmoid	29,746 (5.3)	25,237 (5.3)	2,561 (4.1)	
Small Intestine	15,080 (2.7)	12,156 (2.6)	2,364 (3.8)	
Stomach	42,364 (7.5)	32,313 (6.8)	5,864 (9.4)	
TNM Clinical Stage Group				<0.001
0	19,236 (3.4)	15,818 (3.4)	2,548 (4.1)	
I	145,477 (25.7)	121,883 (25.8)	15,408 (25.0)	
IA	21,686 (3.8)	17,571 (3.7)	2,382 (3.9)	
IB	25,764 (4.6)	21,421 (4.5)	2,719 (4.4)	
IC	7 (0.0)	7 (0.0)	0 (0.0)	
II	18,063 (3.2)	14,520 (3.1)	2,164 (3.5)	
IIA	102,312 (18.1)	86,912 (18.4)	10,288 (16.7)	
IIB	31,539 (5.6)	27,014 (5.7)	2,964 (4.8)	
IIC	5,133 (0.9)	4,334 (0.9)	563 (0.9)	
III	9,643 (1.7)	8,301 (1.8)	799 (1.3)	
IIIA	26,949 (4.8)	22,730 (4.8)	2,677 (4.3)	

© 2022 Bakkila BF et al. *JAMA Network Open*.

IIIB	57,819 (10.2)	48,394 (10.3)	6,214 (10.1)	
**Characteristic**	**All Gastrointestinal Cancer Patients (%)**	**White Gastrointestinal Cancer Patients (%)**	**Black Gastrointestinal Cancer Patients (%)**	***P*-value**
IIIC	18,281 (3.2)	15,397 (3.3)	1,848 (3.0)	
IV	38,165 (6.8)	31,256 (6.6)	4,965 (8.0)	
IVA	29,605 (5.2)	24,089 (5.1)	4,033 (6.5)	
IVB	14,978 (2.7)	12,086 (2.6)	2,130 (3.4)	
IVC	467 (0.1)	400 (0.1)	48 (0.1)	
Grade/Differentiation				<0.001
Well differentiated	83,526 (14.8)	68,367 (14.5)	10,692 (17.3)	
Moderately differentiated	340,088 (60.2)	284,328 (60.2)	37,316 (60.4)	
Poorly differentiated	128,503 (22.7)	108,250 (22.9)	12,621 (20.4)	
Undifferentiated	13,007 (2.3)	11,188 (2.4)	1,121 (1.8)	
**Resection Margins**	**N = 565,124**			
Negative Resection Margins				<0.001
Yes	500,097 (88.5)	417,903 (88.5)	54,321 (88.0)	
No	65,027 (11.5)	54,230 (11.5)	7,429 (12.0)	
**Lymph Nodes**	**N = 492,909**			
Adequate Lymphadenectomy				<0.001
Yes	350,716 (71.2)	294,744 (71.2)	37,661 (70.5)	

© 2022 Bakkila BF et al. *JAMA Network Open*.

No	142,193 (28.8)	119,227 (28.8)	15,766 (29.5)	

[1]"Other" was provided as an option for patient-reported races in the National Cancer Database.

© 2022 Bakkila BF et al. *JAMA Network Open*.

eTable 2. Survival Analysis by Outcome Metric, Race, and Organ Site
Hazard ratios with reference group set as white patients with desired surgical outcome (negative resection margins or adequate lymphadenectomy).

**Organ**	**Hazard Ratios (95% CI)**							
	**Resection Margins**				**Lymphadenectomy**			
	Negative margins		*Positive margins*		*Adequate lymphadenectomy*		*Inadequate lymphadenectomy*	
	White	Black	White	Black	White	Black	White	Black
All organ sites	Ref	1.12 (1.10, 1.14)	2.54 (2.51, 2.57)	2.65 (2.57, 2.71)	Ref	1.13 (1.11, 1.15)	1.10 (1.09, 1.11)	1.20 (1.17, 1.23)
Anus	Ref	1.27 (1.08, 1.50)	1.23 (1.13, 1.34)	1.32 (1.10, 1.58)	Ref	1.09 (0.81, 1.45)	1.07 (0.93, 1.23)	1.22 (0.93, 1.60)
Colon	Ref	1.17 (1.15, 1.20)	3.16 (3.10, 3.21)	3.22 (3.07, 2.27)	Ref	1.14 (1.12, 1.16)	1.23 (1.21, 1.25)	1.47 (1.42, 1.53)
Esophagus	Ref	1.16 (1.05, 1.27)	1.91 (1.81, 2.02)	2.09 (1.66, 2.63)	Ref	1.14 (0.98, 1.33)	1.15 (1.11, 1.19)	1.35 (1.20, 1.51)
Gallbladder	Ref	0.95 (0.86, 1.06)	2.88 (2.70, 3.08)	2.64 (2.32, 3.02)	Ref	1.00 (0.78, 1.29)	1.34 (1.21, 1.49)	1.18 (1.04, 1.34)
Liver	Ref	1.10 (1.03, 1.19)	2.52 (2.35, 2.71)	2.72 (2.27, 3.25)	Ref			
Other Biliary	Ref	1.02 (0.91, 1.15)	2.09 (1.94, 2.26)	2.12 (1.72, 2.60_	Ref			
Pancreas	Ref	1.02 (0.97, 1.07)	1.90 (1.84, 1.95)	1.92 (1.78, 2.07)	Ref	1.07 (1.01, 1.13)	0.89 (0.87, 0.92)	0.87 (0.82, 0.92)
Peritoneum	Ref	0.84 (0.60, 1.17)	1.49 (1.32, 1.67)	1.52 (1.16, 1.99)	Ref			

© 2022 Bakkila BF et al. *JAMA Network Open*.

Organ	Hazard Ratios (95% CI)							
	**Resection Margins**				**Lymphadenectomy**			
	Negative margins		*Positive margins*		*Adequate lymphadenectomy*		*Inadequate lymphadenectomy*	
	White	Black	White	Black	White	Black	White	Black
Rectum	Ref	1.17 (1.12, 1.22)	2.49 (2.41, 2.58)	2.46 (2.23, 2.70)	Ref	1.25 (1.18, 1.32)	1.06 (1.03, 1.09)	1.31 (1.22, 1.40)
Rectosigmoid	Ref	1.35 (1.26, 1.45)	3.26 (3.09, 3.44)	3.49 (2.99, 4.06)	Ref	1.28 (1.19, 1.39)	1.09 (1.04, 1.14)	1.61 (1.43, 1.82)
Small Intestine	Ref	1.24 (1.13, 1.36)	1.77 (1.64, 1.91)	2.00 (1.71, 2.35)	Ref	1.19 (1.05, 1.34)	1.11 (1.04, 1.19)	1.44 (1.28, 1.61)
Stomach	Ref	0.95 (0.91, 1.00)	2.57 (2.47, 2.67)	2.50 (2.29, 2.73)	Ref	0.96 (0.90, 1.02)	0.94 (0.91, 0.98)	0.86 (0.81, 0.91)

© 2022 Bakkila BF et al. *JAMA Network Open*.

eFigure 1. Odds Ratios of Negative Surgical Margins With Covariates (Model 2)
Odds ratios of negative surgical margins, adjusted for the covariates of sex, age group, ethnicity, and organ (not shown) (Model 2).

Variable[1]	OR (95% CI)	P value
**Race**		
White	Reference	
Black	0.96 (0.93, 0.98)	0.001
American Indian	0.89 (0.77, 1.02)	0.086
Asian	1.10 (1.05, 1.16)	<0.001
Pacific Islander	0.86 (0.70, 1.04)	0.117
Other	1.06 (0.96, 1.16)	0.249
Unknown	1.13 (1.02, 1.25)	0.015
**Sex**		
Female	Reference	
Male	1.03 (1.01, 1.05)	0.001
**Age Group**		
40-49	Reference	
50-59	1.11 (1.07, 1.15)	<0.001
60-69	1.11 (1.08, 1.15)	<0.001
70-79	1.13 (1.09, 1.16)	<0.001
80-89	1.07 (1.03, 1.10)	<0.001
90+	0.89 (0.83, 0.95)	<0.001
**Ethnicity**		
Not Hispanic	Reference	
Hispanic	0.94 (0.91, 0.98)	<0.001
Unknown	0.91 (0.88, 0.95)	<0.001

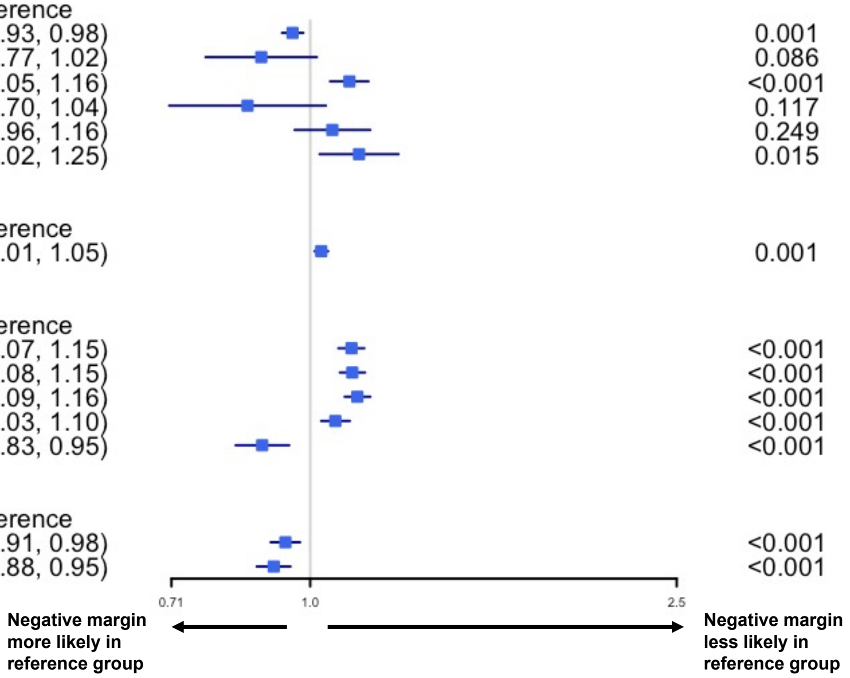

Footnotes:
[1] Model adjusted for sex, age group, ethnicity, and organ site (organ results not shown) (Model 2).

© 2022 Bakkila BF et al. *JAMA Network Open*.

eFigure 2. Odds Ratios of Adequate Lymphadenectomy With Covariates (Model 2)
Odds ratios of adequate lymphadenectomy, adjusted for the covariates of sex, age group, ethnicity, and organ (not shown) (Model 2).

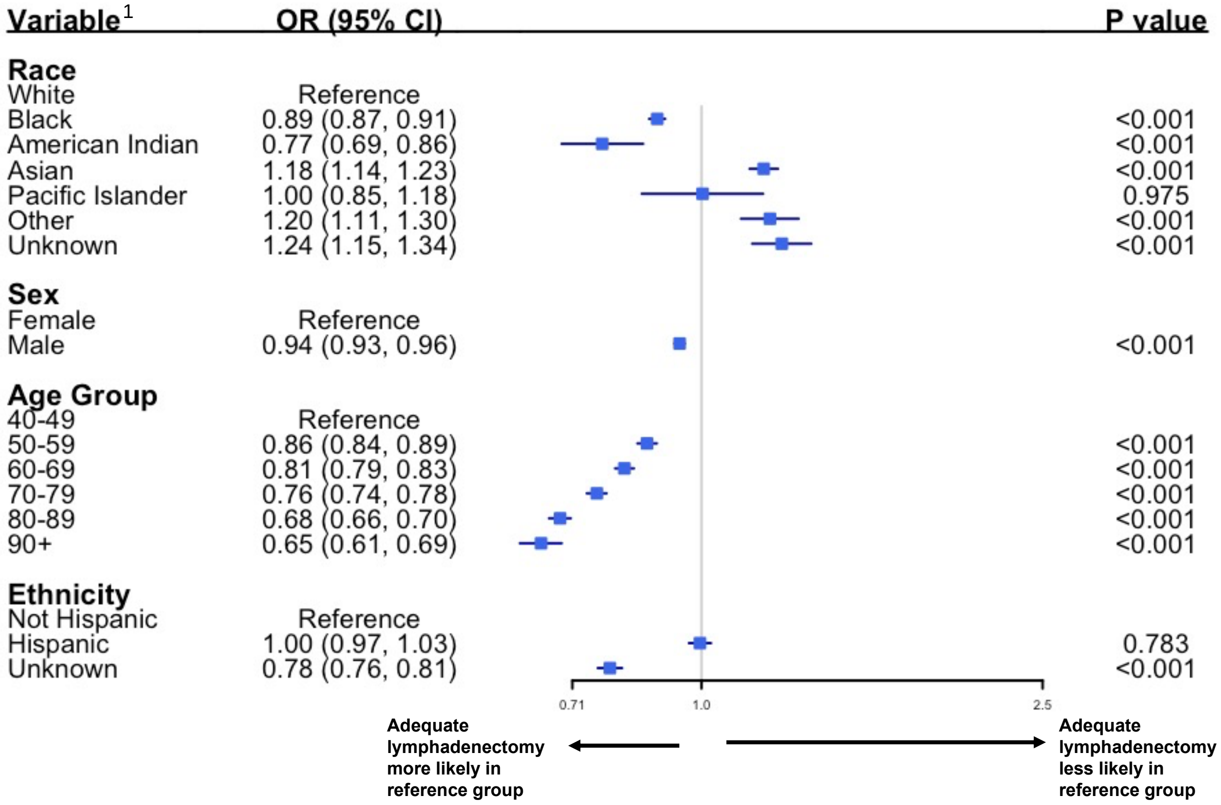

Footnotes:
[1] Model adjusted for sex, age group, ethnicity, and organ site (Model 2).

© 2022 Bakkila BF et al. *JAMA Network Open*.
